# A birth cohort study to investigate the association between prenatal phthalate and bisphenol A exposures and fetal markers of metabolic dysfunction

**DOI:** 10.1186/1476-069X-13-84

**Published:** 2014-10-22

**Authors:** Jillian Ashley-Martin, Linda Dodds, Tye E Arbuckle, Adrienne S Ettinger, Gabriel D Shapiro, Mandy Fisher, Anne-Sophie Morisset, Shayne Taback, Maryse F Bouchard, Patricia Monnier, Renee Dallaire, William D Fraser

**Affiliations:** Perinatal Epidemiology Research Unit, Dalhousie University, Halifax, Nova Scotia Canada; Health Canada, Ottawa, Canada; Yale University, New Haven, CT USA; University of Montreal, Montreal, Quebec Canada; CHU Sainte-Justine Research Centre, Montreal, Quebec Canada; University of Manitoba, Winnipeg, Manitoba Canada; McGill University, Montreal, Quebec Canada; Laval University, Quebec City, Quebec Canada

**Keywords:** Phthalates, Bisphenol A, Leptin, Adiponectin, Pregnancy, Cohort study, Canada

## Abstract

**Background:**

Obesity and type-2 diabetes are on the rise and in utero exposure to environmental contaminants is a suspected contributing factor. Our objective was to examine associations between prenatal exposure to potential endocrine disrupting chemicals and markers of fetal metabolic dysfunction.

**Methods:**

The Maternal-Infant Research on Environmental Chemicals Study (MIREC) recruited 2001 women during the first trimester of pregnancy from 10 Canadian sites. First trimester maternal urine was measured for 11 phthalate metabolites and bisphenol A (BPA). Leptin and adioponectin measured in 1,363 available umbilical cord blood samples served as markers of metabolic function. Restricted cubic spline curves were used to assess the relationship between continuous measures of phthalate and BPA levels and cord blood adipokines. Polytomous logistic regression models were used to estimate odds ratios (OR) and 95% confidence intervals (CI) for the association between phthalates and BPA and both high (≥90th percentile) and low (≤10th percentile) fetal adiponectin and leptin, adjusting for confounding factors. Analyses were conducted for all subjects, overall, and separately by fetal sex.

**Results:**

Leptin was significantly higher in female than male infants. We observed an inverse, non-linear relationship between BPA and adiponectin among males in the restricted cubic spline and linear regression analysis. Mono-(3-carboxypropyl) (MCPP) was associated with increased odds of high leptin among males in the polytomous logistic regression models (4th quartile OR = 3.5 95% CI: 1.1-11.6).

**Conclusion:**

Our findings contribute to the growing body of evidence examining the influence of early life exposure on metabolic regulation and function. Associations between maternal exposure to chemicals and markers of metabolic function appear to be potentially sex specific. However, further investigation is required to determine whether in utero and childhood exposure to BPA and phthalates are associated with metabolic dysfunctions later in life.

## Background

Rates of obesity, diabetes, and metabolic syndrome are on the rise globally and within Canada. Between the years of 1980 and 2008, the global prevalence of obesity doubled from 6.4% to 12.0% [1]. In Canada, nearly 20% of girls and boys between 12–17 years of age were reportedly overweight between 2009 and 2011 [2]. While obesity has been commonly linked to inadequate physical activity and excess caloric intake, recent research suggests that the etiology of the obesity epidemic is multi-factorial [3]. A growing body of evidence has contributed to the hypothesis that exposure to endocrine disruptor chemicals, such as phthalates and bisphenol A (BPA), may operate through complex causal pathways leading from in utero development to childhood and adult obesity. These chemicals are ubiquitous in the environment, have been detected in a majority of the US and Canadian population [4, 5], and have received recent attention for their potential obesogenic properties [6]. Specifically, experimental data has demonstrated that exposure to phthalates and BPA alters normal lipid metabolism and adipogenesis [7]. By binding to PPAR-γ receptors, a critical regulatory component of lipid metabolism and adipogenesis, phthalate exposure has the potential to promote weight gain [7, 8]. BPA exhibits its effects on metabolic function by inducing adipocyte formation, promoting insulin resistance, and inhibiting adiponectin [9, 10].

The fetal time period is a critical window of adipocyte development. Thus, chemical exposures during this time may alter an individual’s growth trajectory and increase risk of later life obesity and metabolic disorders [6–8, 11]. Despite the recognized importance of the in utero environment on child and adult health, detailed understanding of the effect of chemical exposures on newborn endocrine and adipocyte development is lacking [12].

Insight into the susceptibility of fetal development to the potential adverse effects of phthalates and BPA can be obtained by examining biomarkers of fetal metabolic function such as leptin and adiponectin. The physiologic role of these adipocyte-produced hormones in metabolic regulation and function has been recently recognized [13, 14]. Elevated leptin levels in adults are associated with increased body mass index (BMI) [15], and large for gestational age infants [16]. High cord blood leptin levels have been positively correlated with birth weight [17, 18] and insulin resistance [19], whereas low cord blood leptin levels have been associated with small for gestational age [20]. In adults, low adiponectin levels have been implicated in insulin resistance, type 2 diabetes, and metabolic syndrome [21]. Umbilical cord blood adiponectin levels, however, are positively associated with birth weight [21, 22].

While cross sectional studies have demonstrated positive associations between concurrent BPA exposure and anthropometric measures [23, 24], there is limited evidence regarding association between prenatal BPA exposure and cord blood adipokines [25, 26]. Literature regarding the association between phthalates and anthropometric profiles is limited to cross sectional analysis of childhood phthalate exposure and anthropometric measures [27–29] and prospective analysis of prenatal exposures and anthropometric measures at birth [30, 31]. Thus, prospective investigations regarding the potential impact of prenatal exposure to phthalates and BPA on fetal markers of metabolic dysfunction is warranted.

The objective of the present study was to assess the association between prenatal exposure to phthalates and BPA and fetal levels of adiponectin and leptin using data collected in the Maternal-Infant Research on Environmental Chemicals (MIREC) study, a multisite Canadian cohort study.

## Methods

### Study design

Details of the MIREC study have been previously reported [32]. Briefly, 2001 women were recruited from 10 Canadian sites from 2008–2011 during their first trimester of pregnancy and consented to provide urine and blood samples. Women were eligible for inclusion if they were <14 weeks gestation at time of recruitment, ≥18 years of age, able to communicate in French or English, and planning to deliver in a participating hospital. Women with known fetal or chromosomal anomalies in the current pregnancy and women with serious medical complications were excluded from the study [32]. The population in the present investigation included mothers who had a singleton, live, term birth (≥37 weeks) and a cord blood sample suitable for analysis. Pre-term infants were excluded as adiponectin and leptin levels were notably lower prior to 37 weeks gestation as has been previously reported [33].

### Phthalate and bisphenol a exposure

BPA and 11 phthalate metabolites were measured in maternal urine collected during the 1st trimester as shown in Table 1 and as previously described [34]. Briefly, chemical analysis of urine samples was carried out at the Laboratoire de Toxicologie, Institut National de Santé Publique du Québec (Québec, QC, Canada), accredited by the Standards Council of Canada. Phthalates in urine were analyzed by LC-MS/MS with an Ultra Performance Liquid Chromatography (UPLC) coupled with a tandem mass spectrometer and Quattro Premier XE following enzymatic deconjugation. Total BPA in urine was measured with a GC-MS-MS instrument with a GC Agilent 6890 N (Agilent Technologies; Mississauga, ON, Canada) coupled with a tandem mass spectrometer Quattro Micro GC (Waters; Milford, Massachusetts, USA). An enzymatic hydrolysis freed the conjugated compounds in the urine, the samples were then derivatized and the derivatives extracted and analyzed. Specific details on lab methodologies and quality assurance methods have been previously described [35–37].

**Table 1:** **Geometric mean (SD) of phthalate metabolites and bisphenol A by high and low levels of cord blood leptin and adiponectin (MIREC study, n = 1237)**

			Leptin		Adiponectin	
			High (≥90th percentile)	Low (<90th percentile)	High (≥90th percentile)	Low (<90th percentile)
Chemical (μg/L)	LOD	% >LOD	Geometric mean (SD)	Geometric mean (SD)	Geometric mean (SD)	Geometric mean (SD)
Mono-ethyl phthalate (MEP)	0.5	99.8	45.8 (4.2)	37.3 (4.0)	42.1 (3.7)	37.6 (4.1)
Monobutyl phthalate (MBP)	0.2	99.7	12.5 (2.2)	13.23 (2.33)	11.6 (2.2)	13.3 (2.3)
Mono-benzyl phthalate (MBzP)	0.2	99.5	7.1 (2.8)	5.83 (2.67)	5.7 (2.8)	6.0 (2.7)
Mono-(3-carboxypropyl) (MCPP)	0.2	83.9	1.0 (2.9)	0.94 (2.92)	1.0 (3.1)	0.9 (2.9)
Mono-2-ethylhexyl phthalate **(**MEHP)	0.2	98.3	2.6 (2.4)	2.6 (2.5)	2.7 (2.5)	2.6 (2.5)
Mono-(2-ethyl-5-hydroxyhexyl) (MEHHP)	0.4	99.3	10.6 (2.2)	10.6 (2.5)	10.4 (2.5)	10.6 (2.5)
Mono-(2-ethyl-5-oxohexyl) (MEOHP)	0.2	99.7	7.4 (2.1)	7.4 (2.4)	7.1 (2.4)	7.4 (2.3)
Bisphenol A (BPA)	0.2	86.6	0.8 (2.5)	0.9 (2.8)	0.9 (2.5)	0.9 (2.8)
Mono cyclohexyl phthalate (MCHP)	0.2	7.4				
Mono-n-octyl phthalate (MOP)	0.6	3.0				
Mono-isononyl phthalate (MNP)	0.4	1.1				
Mono-methyl phthalate (MMP)	5	14.0				

### Fetal markers of metabolic function

Leptin and adiponectin were measured in plasma from 1363 umbilical cord blood samples. Analysis was done by ELISA at Mt. Sinai Laboratory (Toronto, ON, Canada) using assay kits from Meso Scale Discovery (MSD) (Rockville, MD, USA). Analysis was repeated for all samples with coefficients of variation (CV) greater than 15%. The inter- and intra-assay CVs were 11.8% and 9.3% respectively for leptin and 8% and 9% respectively for adiponectin. All samples were in the range of detection.

### Covariates

Data on covariates were extracted from questionnaires and hospital charts by trained research nurses and staff. We examined the following variables as potential confounders; maternal age at delivery (≤24, 25–29, 30–34 ,≥35 y), pre-pregnancy body mass index (BMI) according to WHO guidelines [38], parity (nulliparous, parous), maternal education (high school diploma or less, some college or trade school, undergraduate university degree, graduate university degree), household income ($ ≤30,000, 30,001-50,000, 50,001-100,000, ≥100,000), ethnicity (Caucasian/non-Caucasian), and maternal smoking (never or quit before pregnancy, quit when knew pregnant, current smoking).

### Statistical analysis

Umbilical cord blood levels of leptin and adiponectin were categorized into the top 90th percentile and bottom 10th percentile. Previous literature has shown that low as well as high levels of both of these biomarkers are associated with potentially adverse outcomes [18, 20, 21]. Due to differing leptin levels among male and female infants, the binary leptin variable was derived using sex specific cut-off points (10th percentile males 1.87 ng/mL, females 3.37 ng/mL; 90th percentile males 31.7 ng/mL, females 59.3 ng/mL). However, as adiponectin levels did not vary by sex, sex-specific cut-offs were not necessary (median for males = 16.7 μg/mL; females = 16.6 μg/mL).

Descriptive statistics for maternal demographics, and pregnancy characteristics were calculated according to levels of leptin and adiponectin using frequency distributions and chi-square tests of significance for the difference between the low (≤10th percentile), moderate (10th-90th percentile) and elevated (≥90th percentile) leptin and adiponectin groups.

Maternal urinary measures of eleven phthalate metabolites and BPA collected from a 1st trimester spot urine sample were available in the MIREC study. Four phthalate metabolites (MCHP, MOP, MNP, MMP) were not examined in multivariate analysis due to the low proportion of values above the limit of detection (LOD) (% > LOD MCHP = 7.4, MOP = 3.0, MNP = 1.1, MMP = 14.0). Three of the phthalates metabolites are primary (MEHP) and secondary (MEHHP, MEOHP) metabolites of the parent compound Di(2-ethylhexyl)phthalate (DEHP) [39]. Considering the high correlation (Pearson correlation coefficient = 0.9) between these metabolites, they were not analyzed individually, but rather the metabolite concentrations were summed to create an index of DEHP metabolite [40].

As the distribution of phthalates and BPA were non-normal, concentrations were log-transformed prior to calculation of descriptive statistics. In order to control for differences in urine dilution, concentrations of phthalate metabolites and BPA were adjusted for specific gravity according to the following formula P_c_ = P_i_ [(SG_m_- 1)/(SG_i_ - 1)] where: *P*_*c*_ = SG adjusted metabolite concentration (μg/ml), *P*_i_ = observed metabolite concentration, *SG*_i_ = specific gravity of the urine sample, and SG_m_ = median SG for the cohort [41]. This formula was applied to phthalate and BPA concentrations in the descriptive statistic analysis but not the multivariate analysis where specific gravity was included as a covariate (described below). Geometric means (GM) and standard deviations (SD) were calculated for the phthalates and BPA according to high and low levels of leptin and adiponectin. Samples below the LOD were imputed as LOD/2 for all analyses.

Given previous evidence of sex differences in the relationship between BPA and adipokines [25], as well as the differences in leptin levels among male and female infants, all analyses were presented for the total population as well as stratified by infant sex. We included variables in the multivariate models that were either selected *a priori* for inclusion or were significantly associated with leptin or adiponectin at a p-value <0.1 level. This strategy was employed to facilitate identification of a common set of potential confounders across all phthalate metabolites and BPA. In the multivariate models, specific gravity was included as a covariate to account for heterogeneity in urinary dilution as previously described [34] and as done in previous analyses of prenatal endocrine disruptor exposures [42, 43]. By including specific gravity as a covariate, we were able to easily examine unadjusted vs. adjusted results. In addition, in order to determine whether the observed associations between the contaminants of interest and adipokines were independent of birth weight, we conducted an analysis adjusting for birth weight z-score.

Restricted cubic spline analysis was performed to examine the nature of relationship between maternal urinary levels of phthalate and BPA exposures and cord blood adipokine levels [44]. Both exposure and outcome variables were log-transformed in this analysis to account for the skewed distribution of the data. Exposures significantly associated with leptin or adiponectin at a p-value <0.05 were further examined in a multivariate generalized linear regression model of log-transformed exposures and outcomes in order to provide a magnitude of effect. Those exposures associated with adipokine levels in a non-linear fashion (p-value < 0.05) were modeled with a quadratic term.

Polytomous logistic regression [45, 46] was employed to examine the relationship between quartiles of environmental chemical exposures and the odds of high (≥90th percentile) and low (≤10th percentile) adiponectin and leptin levels, using those between 10th-90th percentiles as the group without the outcome. These cut-offs were chosen to identify those subjects with notably elevated or depressed adipokine levels. Polytomous logistic regression was used as a feasible means of calculating odds ratios for two outcome groups in comparison to a group without the outcome. In recognition of the rather arbitrary nature of the cut-off points in the polytomous logistic regression, we also conducted a sensitivity analysis with outcome categories defined at the 25th and 75th percentiles (and, similarly, using those between 25th-75th percentiles as the group without the outcome). Of the seven metabolites included in the multivariate statistical analysis, no chemical had more than 25% below the LOD, so all samples below the LOD were included in the lowest quartile of exposure.

This study received ethical approval from the IWK Health Centre (Halifax, NS), Health Canada, and Ste. Justine’s Hospital (Montreal, QC).

## Results

From the 2001 women recruited into the MIREC study, 18 withdrew and asked that all their data and biospecimens be destroyed. Of the remaining 1983 subjects, 1363 infants born to these women had a cord blood sample. Of these 1363 infants, 126 were excluded for multiple birth, pre-term birth, cord blood samples unsuitable for analysis, missing data on all chemicals of interest, or unknown sex resulting in a final sample size of 1237.

Median (IQR) leptin levels (ng/mL) were significantly higher among female infants (16.0 (26.3)) than males (8.7 (13.7)) and ranged from 0.086 to 243. Median (IQR) adiponectin levels (μg/mL) did not differ by sex (males =16.7 (12.9); females =16.6 (12.6)) and ranged from 0.19 to 239. Study population characteristics according to percentiles of leptin and adiponectin are provided in Table 2. Pre-pregnancy BMI, parity, and cord blood adiponectin levels were associated with leptin. Education and cord blood leptin levels were associated with adiponectin (p < 0.1) (Table 2).

**Table 2:** **Maternal and infant characteristics according to percentiles of cord blood leptin (ng/mL) and adiponectin (μg/mL)** ^**a**^

		Leptin (Row%)				Adiponectin (Row%)			
		Percentiles				Percentiles			
Characteristic	N	≤10th	10th-90th	≥90th	p-value	≤10th	10th-90th	≥90th	p-value
*Age (yr)*									
≤24	53	11.3	81.1	7.6	0.50	9.4	81.1	9.4	0.92
25-29	265	9.8	77.7	12.5		10.6	77.7	11.7	
30-34	448	7.8	83.4	8.7		9.4	81.3	9.4	
≥35	472	11.7	78.0	10.4		9.8	80.1	10.2	
*BMI*									
Underweight (<18.5)	27	22.2	70.4	7.4	<0.01	22.2	70.4	7.4	0.22
Normal (18.5 to 24.9)	707	12.8	80.0	7.5		11.0	78.5	10.5	
Overweight (25 to 29.9)	266	3.8	83.8	12.4		8.3	80.8	10.9	
Obese (≥30)	170	5.9	75.9	18.2		7.7	83.5	8.8	
*Parity*									
Nulliparous	520	9.0	77.1	13.9	0.01	9.0	79.6	11.4	0.17
Parous	715	10.5	82.1	7.4		10.6	80.0	9.4	
*Education*									
High school diploma or less	105	10.5	78.1	11.4	0.51	10.5	80.1	8.6	0.10
Some college, or trade school	351	10.3	77.2	12.5		11.7	79.2	9.1	
Undergraduate univ. degree	470	9.8	81.7	8.5		9.2	80.9	10.0	
Graduate university degree	309	9.4	81.2	9.4		9.1	78.6	12.3	
*Household income ($CAD)*									
≤30,000	87	12.6	75.9	11.5	0.50	12.6	79.3	8.1	0.27
30,001-50,000	114	5.3	83.3	11.4		7.9	84.2	7.9	
50,001-100,000	506	10.7	77.9	11.5		11.1	77.3	11.7	
≥100,000	481	9.2	82.3	8.5		8.1	82.1	9.8	
*Ethnicity*									
Caucasian	1064	9.6	80.5	10.0	0.80	10.3	78.8	10.9	0.53
Not Caucasian	173	11.6	77.58	11.0		7.5	86.7	5.8	
*Maternal smoking*									
Never or quit before pregnancy	1091	9.8	80.8	9.4	0.27	9.9	80.0	10.2	0.74
Quit when knew pregnant	86	9.3	75.6	15.1		11.6	80.2	8.1	
Current smoker	60	11.7	73.3	15.3		8.3	78.3	13.3	
*Infant sex*									
Male	677	10.1	79.6	10.3	0.94	9.5	81.7	8.8	0.40
Female	586	9.6	80.5	9.9		10.4	77.7	11.8	
*Cord blood adiponectin (μg/ml)*									
≤10%	123	43.1	50.4	6.5	<0.01	--	--	--	--
10-90%	988	6.7	84.3	9.0					
≥90%	126	2.4	75.4	22.2					
*Cord blood leptin (ng/mL)*									
≤10%	122	--	--	--	--	43.4	54.1	2.5	<0.01
<90%	990					6.3	84.4	9.6	
≥90%	125					6.4	71.2	22.4	

^a^Due to missing covariate data, subgroup totals may not sum to the total sample population.

The geometric mean and standard deviation of the phthalate metabolites and BPA according to high and low levels of leptin and adiponectin are provided in Table 1. Eighty-four subjects were missing data on MEP, MBP, MCPP, MEOHP, and MEHHP, eighty five were missing data on MBzP, and ninety-three were missing data on MEHP. No subjects were missing data on BPA exposure. The Pearson correlation coefficients of log-transformed phthalate metabolites and bisphenol A ranged from 0.3 (between MEP and BPA) to 0.6 (between MBP and DEHP metabolites).

Multivariate leptin models were adjusted for maternal age at delivery, pre-pregnancy BMI, parity, and specific gravity. Multivariable adiponectin models were adjusted for maternal age at delivery, education, and specific gravity.

**Figure 1:**
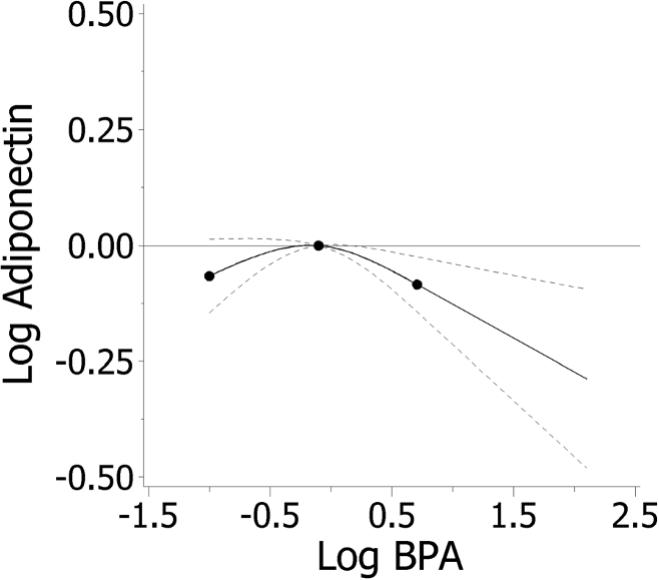
**Restricted cubic spline analysis (95% CI) of log**_**10**_ **BPA and log**_**10**_ **adiponectin among male infants.** Legend *Adjusted for: age, education, specific gravity. Knots indicated by dots at 5th, 50th, and 95th percentiles.

In the restricted cubic spline analysis, maternal urinary BPA concentrations were significantly associated with adiponectin levels (p < 0.05) in a non-linear fashion (p < 0.01) among male but not female infants (Figure 1). In a multivariate regression model, BPA was inversely associated with adiponectin among males (β = −0.02; 95% CI: −0.07-0.03 per one unit increase in BPA). The negative parameter coefficient for the quadratic term (β = −0.10; 95% CI: −0.16- -0.05) is consistent with the slightly inverted U-shape of the spline curve (Figure 1).

In the polytomous logistic regression leptin models, MBzP exposure was associated with an increased odds of high leptin, particularly in the third quartile (OR = 1.8; 95% CI: 0.9-3.7) (Table 3). The third quartile of MBzP exposure was associated with an elevated odds of high leptin among females (OR = 2.7 95% CI: 0.9-7.8). The association among females became significant after adjustment for birth weight z-score (OR = 3.2 95% CI: 1.1-9.5). This effect estimate indicates that subjects with moderately elevated levels of maternal MBzP exposure had increased odds of having high leptin levels relative to those individuals with low MBzP exposure. Subjects with moderate adipokine levels make up the group without the outcome in the polytomous regression analysis. MCPP exposure was associated with a significantly increased odds of high leptin in all quartiles among males only (4th quartile OR = 3.5; 95% CI: 1.1-11.6) (Table 3). There were no significant associations between any of the other phthalate metabolites in the leptin models. Other than the change noted in the MBzP model, adjustment for birth weight z-score for these results did not produce any notable changes in significance or direction of effect. Effect estimates in the sensitivity analyses of the 25th and 75th percentiles tended to attenuate effects toward the null. However, the highest quartile of MCPP exposure remained significantly associated with high leptin (OR = 2.2; 95% CI: 1.1-4.4).

**Table 3:** **Prenatal exposure to phthalate metabolites (quartiles) and odds of low (≤10th percentile) and high (≥90th percentile) and cord blood leptin levels**

	Low leptin ^a^		High leptin ^a^	
Phthalate metabolites	Unadjusted OR	Adjusted OR ^b^	Unadjusted OR	Adjusted OR ^b^
	(95% CI)	(95% CI)	(95% CI)	(95% CI)
MEP				
Total (n =1089)				
≤11	1.0	1.0	1.0	1.0
12- ≤ 28	0.8 (0.5-1.3)	0 8 (0.5-1.4)	0.7 (0.4-1.4)	0.7 (0.4-1.3)
29- ≤ 82	0.5 (0.3-1.0)	0.5 (0.3-1.0)	1.1 (0.7-2.0)	1.1 (0.6-2.1)
>82	0.6 (0.3-1.0)	0.6 (0.3-1.0)	1.0 (0.6-1.8)	0.9 (0.5-1.7)
Males (n = 578)				
≤11	1.0	1.0	1.0	1.0
12- ≤ 28	0.7 (0.3-1.4)	0.7 (0.3-1.4)	0.9 (0.4-2.1)	0.9 (0.4-2.2)
29- ≤ 82	0.4 (0.2-0.9)	0.4 (0.2-0.9)	1.4 (0.7-3.1)	1.6 (0.7-3.7)
>82	0.6 (0.3-1.3)	0.6 (0.3-1.4)	1.4 (0.6-3.0)	1.4 (0.6-3.4)
Females (n = 511)				
≤11	1.0	1.0	1.0	1.0
12- ≤ 28	1.0 (0.5-2.0)	1.0 (0.5-2.4)	0.7 (0.3-1.7)	0.7 (0.3-1.6)
29- ≤ 78	0.7 (0.3-1.6)	0.7 (0.3-1.8)	1.1 (0.5-2.4)	1.1 (0.5-2.5)
>78	0.5 (0.2-1.2)	0.6 (0.2-1.5)	0.7 (0.3-1.6)	0.6 (0.2-1.6)
MBP				
Total (n =1089)				
≤4.9	1.0	1.0	1.0	1.0
>5- ≤ 12	0.6 (0.3-1.0)	0.6 (0.3-1.1)	1.0 (0.6-1.8)	1.0 (0.5-1.8)
13- ≤ 23	0.5 (0.3-0.9)	0.6 (0.3-1.3)	1.0 (0.6-1.8)	0.9 (0.4-1.9)
>23	0.9 (0.5-1.5)	1.0 (0.5-2.3)	1.0 (0.5-1.7)	0.8 (0.4-1.9)
Males (n =578)				
≤5	1.0	1.0	1.0	1.0
>5- ≤ 12	0.9 (0.4-1.8)	1.0 (0.4-2.2)	1.3 (0.6-2.8)	1.2 (0.5-2.8)
13- ≤ 23	0.4 (0.2-1.0)	0.6 (0.2-1.7)	1.1 (0.5-2.3)	0.9 (0.4-2.5)
>23	1.2 (0.6-2.4)	1.9 (0.7-5.5)	0.8 (0.3-1.9)	0.6 (0.2-2.1)
Females (n =511)				
≤4.8	1.0	1.0	1.0	1.0
>4.9- ≤ 11.8	0.4 (0.2-1.0)	0.7 (0.3-1.5)	0.9 (0.4-2.0)	0.9 (0.4-2.3)
11.9- ≤ 23	0.6 (0.3-1.2)	0.8 (0.3-2.3)	0.9 (0.4-2.1)	1.0 (0.3-2.9)
>23	0.6 (0.3-1.4)	0.8 (0.2-2.5)	1.1 (0.5-2.5)	1.2 (0.4-4.0)
MBzP				
Total (n =1088)				
≤2.2	1.0	1.0	1.0	1.0
2.3- ≤ 5.0	0.7 (0.4-1.2)	0.7 (0.4-1.3)	1.5 (0.8-2.8)	1.6 (0.8-3.1)
5.1- ≤ 11.0	0.8 (0.5-1.4)	0.9 (0.5-1.7)	1.6 (0.9-3.0)	1.8 (0.9-3.7)
>11	0.7 (0.4-1.2)	0.8 (0.4-1.7)	1.5 (0.8-2.8)	1.7 (0.8-3.6)
Males (n =577)				
≤2.2	1.0	1.0	1.0	1.0
2.3- ≤ 5.0	0.9 (0.4-2.0)	1.1 (0.5-2.4)	1.6 (0.7-3.5)	1.5 (0.7-3.6)
5.1- ≤ 11.0	0.8 (0.4-1.7)	0.9 (0.4-2.3)	1.4 (0.6-3.2)	1.7 (0.7-4.2)
>11	0.9 (0.4-1.9)	1.1 (0.4-2.8)	1.3 (0.6-2.9)	1.4 (0.5-3.9)
Females (n = 511)				
≤2.2	1.0	1.0	1.0	1.0
2.3- ≤ 5.0	0.6 (0.2-1.2)	0.6 (0.3-1.4)	1.8 (0.7-4.7)	2.2 (0.8-5.8)
5.1- ≤ 11.0	0.9 (0.4-1.9)	1.1 (0.4-2.9)	2.3 (0.9-5.9)	2.7 (0.9-7.8)
>11	0.6 (0.2-1.3)	0.8 (0.3-2.3)	1.9 (0.8-5.0)	2.3 (0.7-7.2)
MCPP				
Total (n =1089)				
≤0.28	1.0	1.0	1.0	1.0
0.29- ≤ 0.85	0.7 (0.4-1.2)	0.5 (0.3-1.0)	1.6 (0.9-2.9)	1.7 (0.9-3.2)
0.86- ≤ 2.0	0.5 (0.3-0.9)	0.5 (0.3-1.0)	1.4 (0.7-2.5)	1.6 (0.8-3.2)
>2.0	0.7 (0.4-1.3)	0.7 (0.4-1.5)	1.5 (0.8-2.7)	1.7 (0.8-3.8)
Males (n =578)				
≤0.29	1.0	1.0	1.0	1.0
0.3- ≤ 0.87	0.9 (0.4-1.8)	0.8 (0.4-1.7)	3.6 (1.4-9.4)	4.3 (1.6-11.6)
0.88- ≤ 2.0	0.3 (0.1-0.7)	0.3 (0.1-0.7)	2.7 (1.0-7.1)	3.6 (1.2-10.8)
>2.0	0.8 (0.4-1.6)	0.7 (0.3-1.7)	2.4 (0.9-6.5)	3.5 (1.1-11.6)
Females (n =511)				
≤0.29	1.0	1.0	1.0	1.0
0.3- ≤ 0.82	0.5 (0.2-1.1)	0.5 (0.2-1.2)	1.2 (0.5-2.7)	1.2 (0.5-3.0)
0.83- ≤ 1.8	0.8 (0.4-1.8)	1.1 (0.4-2.7)	0.9 (0.4-2.2)	1.0 (0.4-2.8)
>1.8	0.6 (0.3-1.4)	0.9 (0.3-2.8)	1.3 (0.6-3.0)	1.3 (0.4-4.1)
∑ DEHP				
Total (n =1080)				
≤7.9	1.0	1.0	1.0	1.0
8.0- ≤ 18.1	0.8 (0.5-1.4)	0.8 (0.5-1.5)	1.3 (0.7-2.4)	1.4 (0.7-2.6)
18.2- ≤ 36.0	0.8 (0.4-1.4)	0.8 (0.4-1.7)	1.6 (0.9-2.8)	1.8 (0.9-3.6)
>36.0	0.8 (0.4-1.3)	0.9 (0.4-2.0)	0.9 (0.5-1.7)	1.0 (0.4-2.3)
Males (n =576)				
≤7.9	1.0	1.0	1.0	1.0
8.0-- ≤ 18.1	1.3 (0.6-2.8)	1.4 (0.6-3.2)	1.3 (0.6-2.8)	1.3 (0.5-2.9)
18.2- ≤ 36.0	1.1 (0.5-2.4)	1.3 (0.5-3.2)	1.3 (0.6-2.8)	1.3 (0.5-3.5)
>36.0	0.8 (0.4-1.8)	1.0 (0.3-2.9)	0.6 (0.3-1.4)	0.5 (0.2-1.8)
Females (n =504)				
≤7.4	1.0	1.0	1.0	1.0
7.5-- ≤ 16.4	0.5 (0.2-1.2)	0.5 (0.2-1.3)	1.5 (0.6-3.8)	1.7 (0.7-4.6)
16.4- ≤ 33.0	0.5 (0.2-1.2)	0.5 (0.2-1.4)	1.8 (0.8-4.5)	2.3 (0.8-6.7)
>33.0	0.7 (0.3-1.4)	0.7 (0.2-2.2)	1.5 (0.6-3.8)	2.0 (0.6-7.1)

^a^Moderate adipokine levels (10-90th percentile) represent those without the outcome.

^b^Adjusted for: maternal age, pre-pregnancy BMI, specific gravity, parity.

In the polytomous logistic regression adiponectin models, the third quartile of MCPP exposure was associated with a significantly reduced odds of low adiponectin among males only (OR = 0.3; 95% CI: 0.1-0.7) (Table 4). BPA was significantly inversely associated with high adiponectin in the third quartile of the unadjusted analysis only (OR = 0.3; 95% CI: 0.1-0.8) (Table 5). As with the leptin models, these results were not changed by adjustment for birth weight z-score and results were slightly attenuated towards the null in the sensitivity analysis. There were no significant associations between cord blood leptin levels and prenatal exposure to BPA (Table 5).

**Table 4:** **Prenatal exposure to phthalate metabolites (quartiles) and odds of low (≤10th percentile) and high (≥90th percentile) cord blood adiponectin levels, overall and by sex**

	Low adiponectin ^a^		High adiponectin ^a^	
Phthalate metabolites	Unadjusted OR	Adjusted OR ^b^	Unadjusted OR	Adjusted OR ^b^
	(95% CI)	(95% CI)	(95% CI)	(95% CI)
MEP				
Total (n =1148)				
≤11	1.0	1.0	1.0	1.0
12- ≤ 28	1.1 (0.6-1.8)	1.2 (0.7-2.0)	0.8 (0.5-1.4)	1.1 (0.6-1.9)
29- ≤ 82	0.7 (0.4-1.3)	0.8 (0.4-1.5)	0.9 (0.5-1.5)	1.2 (0.7-2.2)
>82	1.1 (0.6-1.9)	1.2 (0.7-2.2)	0.9 (0.5-1.6)	1.5 (0.8-2.7)
Males (n = 608)				
≤11	1.0	1.0	1.0	1.0
12- ≤ 28	1.2 (0.5-2.5)	1.3 (0.6-2.9)	0.9 (0.4-2.0)	1.3 (0.5-3.0)
29- ≤ 82	1.0 (0.4-2.1)	0.9 (0.4-2.2)	1.0 (0.4-2.1)	1.6 (0.7-3.8)
>82	1.2 (0.6-2.6)	1.4 (0.6-3.3)	0.9 (0.4-2.1)	1.7 (0.7-4.3)
Females (n = 540)				
≤11	1.0	1.0	1.0	1.0
12- ≤ 28	1.1 (0.6-2.3)	1.2 (0.5-2.5)	1.1 (0.5-2.3)	1.3 (0.6-2.8)
29- ≤ 82	0.5 (0.2-1.2)	0.9 (0.4-2.0)	0.7 (0.3-1.5)	0.9 (0.4-2.0)
>82	1.1 (0.5-2.3)	1.1 (0.5-2.6)	1.2 (0.6-2.5)	1.7 (0.8-3.9)
MBP				
Total (n =1148)				
≤5	1.0	1.0	1.0	1.0
>5- ≤ 12	0.6 (0.3-1.0)	0.6 (0.3-1.1)	1.0 (0.6-1.6)	1.1 (0.7-1.9)
13- ≤ 23	0.5 (0.3-0.8)	0.5 (0.3-1.0)	0.4 (0.3-0.8)	0.6 (0.3-1.2)
>23	1.0 (0.6-1.6)	1.0 (0.4-2.2)	0.6 (0.4-1.1)	0.9 (0.4-2.1)
Males (n =608)				
≤5	1.0	1.0	1.0	1.0
>5- ≤ 12	0.6 (0.3-1.2)	0.6 (0.3-1.4)	0.9 (0.4-1.8)	0.8 (0.4-1.8)
13- ≤ 23	0.4 (0.2-1.0)	0.5 (0.2-1.3)	0.5 (0.2-1.0)	0.4 (0.2-1.2)
>23	1.0 (0.5-1.9)	1.0 (0.4-3.1)	0.2 (0.1-0.6)	0.2 (0.1-0.8)
Females (n =540)				
≤5	1.0	1.0	1.0	1.0
>5- ≤ 12	0.7 (0.3-1.4)	0.7 (0.3-1.6)	1.1 (0.5-2.2)	1.3 (0.6-2.8)
13- ≤ 23	0.6 (0.3-1.2)	0.6 (0.2-1.6)	0.4 (0.2-1.0)	0.7 (0.2-1.9)
>23	0.9 (0.5-1.9)	1.0 (0.4-3.0)	1.1 (0.5-2.3)	2.1 (0.8-5.7)
MBzP				
Total (n =1147)				
≤2.2	1.0	1.0	1.0	1.0
2.3- ≤ 5.0	1.0 (0.6-1.8)	1.1 (0.6-1.9)	1.1 (0.7-1.9)	1.4 (0.8-2.3)
5.1- ≤ 11.0	0.8 (0.4-1.3)	0.8 (0.4-1.6)	0.7 (0.4-1.2)	1.0 (0.5-1.9)
>11	1.0 (0.6-1.7)	1.0 (0.5-2.0)	0.7 (0.4-1.2)	1.1 (0.6-2.3)
Males (n =607)				
≤2.2	1.0	1.0	1.0	1.0
2.3- ≤ 5.0	1.3 (0.6-2.9)	1.4 (0.6-3.2)	1.2 (0.6-2.5)	1.5 (0.7-3.2)
5.1- ≤ 11.0	0.9 (0.4-2.0)	1.0 (0.4-2.5)	0.7 (0.3-1.5)	1.0 (0.4-2.5)
>11	1.2 (0.6-2.6)	1.4 (0.5-3.6)	0.3 (0.1-0.9)	0.5 (0.2-1.7)
Females (n = 540)				
≤2.2	1.0	1.0	1.0	1.0
2.3- ≤ 5.0	0.8 (0.4-1.7)	0.8 (0.4-1.8)	1.0 (0.5-2.1)	1.2 (0.6-2.6)
5.1- ≤ 11.0	0.7 (0.3-1.5)	0.7 (0.3-1.7)	0.7 (0.3-1.5)	1.0 (0.4-2.5)
>11	0.8 (0.4-1.7)	0.8 (0.3-2.1)	1.1 (0.5-2.2)	1.9 (0.7-4.8)
MCPP				
Total (n =1148)				
≤0.29	1.0	1.0	1.0	1.0
0.3- ≤ 0.87	0.9 (0.5-1.5)	0.9 (0.5-1.5)	1.3 (0.8-2.3)	1.6 (0.9-2.7)
0.88- ≤ 2.0	0.6 (0.4-1.1)	0.6 (0.3-1.1)	0.7 (0.4-1.3)	1.1 (0.6-2.2)
>2.0	0.8 (0.4-1.3)	0.7 (0.3-1.4)	0.9 (0.5-1.5)	1.6 (0.8-3.4)
Males (n =608)				
≤0.29	1.0	1.0	1.0	1.0
0.3- ≤ 0.87	1.0 (0.5-1.9)	0.9 (0.4-1.8)	1.3 (0.6-2.7)	1.5 (0.7-3.2)
0.88- ≤ 2.0	0.4 (0.2-0.9)	0.3 (0.1-0.7)	0.5 (0.2-1.2)	0.8 (0.3-2.2)
>2.0	0.7 (0.3-1.4)	0.4 (0.2-1.2)	0.6 (0.3-1.4)	1.1 (0.4-3.1)
Females (n =540)				
≤0.29	1.0	1.0	1.0	1.0
0.3- ≤ 0.87	0.8 (0.4-1.7)	0.8 (0.4-1.8)	1.6 (0.7-3.3)	1.9 (0.9-4.2)
0.88- ≤ 2.0	0.9 (0.4-1.8)	0.9 (0.4-2.2)	1.1 (0.5-2.4)	1.8 (0.7-4.5)
>2.0	0.8 (0.4-1.7)	0.9 (0.3-2.5)	1.3 (0.6-2.8)	2.9 (1.0-7.8)
∑ DEHP				
Total (n =1139)				
≤7.9	1.0	1.0	1.0	1.0
8.0- ≤ 18.1	1.1 (0.6-1.8)	1.1 (0.6-1.9)	0.6 (0.3-1.0)	0.7 (0.4-1.3)
18.2- ≤ 36.0	1.2 (0.7-2.0)	1.2 (0.6-2.3)	0.7 (0.4-1.2)	1.1 (0.6-2.0)
>36.0	0.6 (0.4-1.2)	0.7 (0.3-1.5)	0.6 (0.4-1.1)	1.1 (0.5-2.4)
Males (n =606)				
≤7.9	1.0	1.0	1.0	1.0
8.0- ≤ 18.1	1.5 (0.7-3.3)	1.6 (0.7-3.6)	0.6 (0.3-1.4)	0.8 (0.4-1.9)
18.2- ≤ 36.0	1.3 (0.6-2.9)	1.5 (0.6-3.8)	0.6 (0.3-1.3)	1.0 (0.4-2.6)
>36.0	0.8 (0.4-2.0)	0.9 (0.3-2.9)	0.6 (0.3-1.2)	1.2 (0.4-3.5)
Females (n =533)				
≤7.9	1.0	1.0	1.0	1.0
8.0- ≤ 18.1	0.6 (0.3-1.4)	0.6 (0.3-1.3)	0.9 (0.4-1.9)	1.0 (0.4-2.1)
18.2- ≤ 36.0	0.9 (0.4-1.8)	0.7 (0.3-1.8)	0.7 (0.4-1.6)	1.0 (0.4-2.5)
>36.0	0.5 (0.2-1.1)	0.3 (0.1-1.1)	0.7 (0.3-1.5)	1.2 (0.4-3.4)

^a^Moderate adipokine levels (10-90th percentile) represent those without the outcome.

^b^Adjusted for: maternal age, specific gravity, education.

**Table 5:** **Prenatal exposure to bisphenol A (quartiles) and odds low (≤10th percentile) and high (≥90th percentile) cord blood adipokines, overall and by sex**

	Low leptin ^a^		High leptin ^a^	
Contaminant	Unadjusted OR	Adjusted OR ^b^	Unadjusted OR	Adjusted OR ^b^
	(95% CI)	(95% CI)	(95% CI)	(95% CI)
BPA				
Total (n =1165)				
≤0.34	1.0	1.0	1.0	1.0
0.35- ≤ 0.81	1.2 (0.7-2.0)	1.3 (0.8-2.3)	0.9 (0.5-1.5)	0.9 (0.5-1.6)
0.82- ≤ 1.7	1.1 (0.6-1.8)	1.3 (0.7-2.5)	1.2 (0.7-2.0)	1.1 (0.6-2.0)
>1.7	0.9 (0.5-1.6)	1.3 (0.7-2.6)	1.1 (0.6-1.8)	0.9 (0.5-1.8)
Males (n =626)				
≤0.34	1.0	1.0	1.0	1.0
0.35- ≤ 0.81	0.9 (0.4-2.0)	1.0 (0.5-2.3)	1.1 (0.5-2.3)	1.0 (0.5-2.3)
0.82- ≤ 1.7	1.0 (0.5-2.0)	1.1 (0.5-2.6)	1.2 (0.6-2.6)	1.2 (0.5-2.9)
>1.7	0.8 (0.4-1.6)	1.0 (0.4-2.4)	1.2 (0.6-2.4)	1.0 (0.4-2.4)
Females (n =539)				
≤0.34	1.0	1.0	1.0	1.0
0.35- ≤ 0.81	1.6 (0.3-1.6)	1.8 (0.8-4.1)	0.7 (0.3-1.6)	0.7 (0.3-1.6)
0.82- ≤ 1.7	1.2 (0.5-2.8)	1.6 (0.6-4.4)	1.1 (0.5-2.3)	1.0 (0.4-2.4)
>1.7	1.1 (0.5-2.7)	1.8 (0.6-5.1)	1.0 (0.5-2.2)	0.8 (0.3-2.2)
	**Low adiponectin** ^**a**^		**High adiponectin** ^**a**^	
	**Unadjusted OR**	**Adjusted OR** ^**c**^	**Unadjusted OR**	**Adjusted OR** ^**c**^
	**(95% CI)**	**(95% CI)**	**(95% CI)**	**(95% CI)**
BPA				
Total (n =1232)				
≤0.34	1.0	1.0	1.0	1.0
0.35- ≤ 0.81	0.8 (0.5-1.4)	0.9 (0.5-1.5)	0.7 (0.4-1.2)	0.8 (0.5-1.4)
0.82- ≤ 1.7	0.7 (0.4-1.3)	0.8 (0.4-1.4)	0.7 (0.4-1.1)	1.0 (0.5-1.8)
>1.7	0.8 (0.5-1.4)	0.9 (0.4-1.6)	0.6 (0.4-1.1)	1.0 (0.5-1.9)
Males (n =660)				
≤0.34	1.0	1.0	1.0	1.0
0.35- ≤ 0.81	0.9 (0.4-1.8)	0.9 (0.4-1.9)	0.6 (0.3-1.3)	0.8 (0.3-1.6)
0.82- ≤ 1.7	0.7 (0.3-1.5)	0.7 (0.3-1.7)	0.3 (0.1-0.8)	0.5 (0.2-1.2)
>1.7	0.8 (0.4-1.7)	0.8 (0.3-2.0)	0.8 (0.4-1.7)	0.9 (0.4-2.2)
Females (n =572)				
≤0.34	1.0	1.0	1.0	1.0
0.35- ≤ 0.81	0.8 (0.4-1.7)	0.9 (0.4-1.9)	0.8 (0.4-1.6)	0.9 (0.4-1.9)
0.82- ≤ 1.7	0.8 (0.3-1.6)	0.8 (0.3-2.0)	1.1 (0.6-2.3)	1.7 (0.8-3.8)
>1.7	0.8 (0.4-1.7)	0.9 (0.3-2.2)	0.7 (0.3-1.5)	1.1 (0.5-2.8)

^a^Moderate adipokine levels (10-90th percentile) represent those without the outcome.

^b^Adjusted for: maternal age, pre-pregnancy BMI, specific gravity, parity.

^c^Adjusted for: maternal age, specific gravity, education.

## Discussion

In this longitudinal birth cohort study of Canadian women, we evaluated the relationship between maternal urinary levels of phthalate and BPA and umbilical cord blood levels of leptin and adiponectin. We observed an inverse, non-linear relationship between BPA and adiponectin among males in the restricted cubic spline and linear regression analysis. We also observed a significantly increased risk of high (≥90th percentile) leptin among male subjects with moderate and elevated MCPP maternal exposure levels. The relationship between MCPP and leptin, however, was not significant in the spline analysis (p = 0.2) and was attenuated towards the null in the sensitivity analysis. Moreover, the wide confidence intervals in some analyses indicate a high level of imprecision in the observed odds ratios.

One previous study reported that prenatal BPA exposure was associated with changes in fetal cord blood adipokine levels [25] with contrasted findings depending on infant’s sex. Authors of this study reported that the highest quartile of maternal BPA exposure was significantly associated with odds of low adiponectin, positively in males and negatively in females and with increased odds of high cord blood leptin levels both in males and females [25]. The authors report a U-shaped relationship between BPA and leptin among females in this study and there was no clear dose–response relationship among males. High adiponectin was not considered as an outcome in this study [25]. Whereas the MIREC cohort is a primarily Caucasian Canadian population, the Chou birth cohort is Taiwanese [25]. Genetic differences in metabolism between different ethnic groups may be a partial explanation for the varying findings. Further, Chou et al. [25] measured BPA in plasma (GM = 2.5, range 0.3-29.4 ng/mL) while MIREC used urine, which makes comparisons difficult. Therefore, differences in the study population and sample matrix used for BPA analysis may account for the differences in results between our study and the study by Chou et al. [25].

Though minimizing contamination in BPA analysis necessitates stringent quality assurance practices in all analyses [47], urinary measurements of BPA are thought to be more robust to contamination than plasma or serum [48]. Fetal levels of adiponectin in the present analysis (18.23 μg/mL) and as reported by Chou et al. (21.3 ug/mL) [25] were similar. However, mean leptin levels in the present analysis (19.8 ng/mL) were notably higher than the mean level of 4.6 ng/mL reported in the Taiwanese cohort [25]. The reason for the discrepancy in leptin levels is not clear. It is not explained by mean pre-pregnancy BMI, which was comparable in the Taiwanese study (25.2) and the MIREC study (24.9), nor was it due to differences in analytical technique as both studies used cord blood plasma analyzed by ELISA. MIREC cord blood leptin levels are, however, comparable to those reported in a pregnancy cohort from Montreal, Canada (mean leptin = 39.8 ng/mL) [49].

Though no previous prospective analysis of the relationship between prenatal phthalate exposure and fetal adipokine levels has been reported, previous birth cohort studies in Japan [30] and New York city [31] and a French case–control study [50] have reported no significant associations or monotonic relationships between prenatal phthalate exposure and size at birth. Previous cross-sectional analysis of the association between childhood phthalate levels and anthropometric measures have reported both positive associations [28, 29] and no association in children [27]. Though there was no association between childhood urinary levels of any phthalate metabolite studied and BMI in the NHANES cross sectional analysis from 1999–2002, a significant dose–response relationship between quartiles of MBzP levels among males ages 20–59 and BMI was observed [27]. Maternal urinary MBzP and MEP levels in the MIREC cohort (MBzP GM = 5.94 μg/l, MEP GM = 38.0 μg/l) are lower than those reported among US pregnant women from the 2003–2004 NHANES study (MBzP GM = 15.12 μg/l, MEP GM = 79.0 μg/l) [4]. The lower levels observed in the MIREC study are consistent with a temporal trend towards decreasing levels of a subset of phthalates in the North American population [51].

Interpretation of our findings warrants consideration of the trajectory of these adipokines from birth to childhood. Leptin concentrations tend to either remain stable or to increase with age [52]. Thus, a trend towards high leptin levels at birth could potentially translate into adverse metabolic outcomes at a later age. In contrast to leptin, adiponectin levels are higher at birth than in childhood and adulthood and lower levels are associated with greater risk of metabolic disorders in adulthood [21]. In a recent analysis of adipokine levels from birth to childhood, Volberg et al. [52] identified three different adiponectin trajectories; stable, moderately decreasing, and rapidly decreasing then rebounding. Thus, further exploration of the determinants of fetal and childhood adiponectin trajectories and resulting childhood anthropometric measures will help elucidate the implications of elevated adiponectin levels in cord blood.

One of the primary strengths of this study was the rich covariate and exposure data collected prospectively in the MIREC study. We were able to control for notable confounders and were not reliant on proxy measures of exposure, such as self-reported use of products containing phthalates. In addition, the MIREC study population was drawn from 10 different sites across Canada and thus represents some of the diverse geographic regions of the country. Furthermore, the study sample size is larger than previous prospective cohort analysis of the association between prenatal exposures and fetal adipokines.

Despite these strengths, this study has some limitations common to observational studies. First, the use of one measure of exposure during the 1st trimester of pregnancy assumes that this measurement is representative of exposure during the critical window of adipocyte development and regulation. Yet, due to the short half life and rapid elimination of these chemicals, the body burden of phthalates and BPA may vary on a daily basis [53]. Though this potential misclassification would most likely be non-differential, it is not possible to completely predict the direction of resulting bias on the effect estimates. In pregnant women, the reported intraclass correlation (ICCs) for BPA has been very low, ranging from 0.11-0.24 [54–57]. Phthalate metabolites MBzP and MBP were reported to be moderately reproducible based on an ICC of 0.41 and 0.42, respectively, for measures in samples collected at three time points in pregnant women [58]. Second, though the MIREC study had a rich set of covariate data, the role of residual confounding in our findings cannot be ruled out. Third, due to the number of comparisons conducted in this analysis, it is possible that a statistically significant finding was identified by chance. However, due to the lack of previous literature in this area and to the hypothesis generating nature of this investigation, we decided to examine each individual chemical in a separate model and not adjust for multiple comparisons. Fourth, considering that our analysis was restricted to the subset of women with cord blood samples, it is possible that our results are subject to potential selection bias. Since, however, distribution of key characteristics (e.g. age, weight, exposure levels) were similar between our analytic sample and the full study population, it is unlikely that this posed a material threat to internal validity. Last, we cannot rule out the influence of unmeasured co-exposures, such as other endocrine disrupting chemicals, on the relationship between phthalates, BPA and cord blood adipokine levels. Furthermore, considering that the MIREC study population is largely Caucasian, more educated and of a higher income group than the Canadian population at large, generalization to other ethnic and socioeconomic populations should be done with caution.

## Conclusions

This prospective longitudinal cohort study is one of the first to examine associations between prenatal exposures to common environmental contaminants and fetal measures of metabolic function. Our findings contribute to the growing body of evidence examining the influence of early life exposure on metabolic regulation and function [59, 60]. Further investigation is required to determine whether in utero and childhood exposure to these endocrine disrupting chemicals are associated with metabolic dysfunctions later in life. Further investigation is also required to enhance understanding of the potential sex-specific nature of the association between certain chemicals and markers of metabolic function. The need for such follow-up is evidenced by findings from a longitudinal study that reported significant associations between maternal urinary BPA levels measured during pregnancy and blood leptin levels in nine year old children [26]. Future follow-up of the MIREC cohort will facilitate exploration of the associations between maternal urinary contaminant levels and childhood growth measures.

## Abbreviations

BPA: Bisphenol A
MIREC: Maternal-Infant Research on Environmental Chemicals
ELISA: Enzyme linked immunoassay
CV: Coefficients of variations
BMI: Body mass index
WHO: World Health Organization
MCHP: Mono cyclohexyl phthalate
MOP: Mono-n-octyl phthalate
MNP: Mono-isononyl phthalate
MMP: Mono-methyl phthalate
MEHP: Mono-2-ethylhexyl phthalate
MEHHP: Mono-(2-ethyl-5-hydroxyhexyl)
MEOHP: Mono-(2-ethyl-5-oxohexyl)
DEHP: Di(2-ethyl hexyl) phthalate
GM: Geometric mean
SD: Standard deviation
LOD: Limit of detection
IQR: Interquatile range
ICC: Intraclass correlation.
